# Effects of screening coverage and screening quality assurance on cervical cancer mortality: Implication for integrated framework to monitor global implementation of cervical cancer screening programmes

**DOI:** 10.7189/jogh.14.04189

**Published:** 2024-10-25

**Authors:** Minmin Wang, Hongda Chen, Martin C S Wong, Junjie Huang, Yinzi Jin, Zhi-Jie Zheng

**Affiliations:** 1 Department of Global Health, School of Public Health, Peking University, Beijing, China; 2 Institute for Global Health and Development, Peking University, Beijing, China; 3 Medical Research Centre, Peking Union Medical College Hospital, Chinese Academy of Medical Sciences and Peking Union Medical College, Beijing, China; 4 The Jockey Club School of Public Health and Primary Care, Faculty of Medicine, The Chinese University of Hong Kong, Hong Kong, China

## Abstract

**Background:**

Cervical cancer is a global health threat and a manifestation of inequality, and screening is an effective intervention. However, little is known about how screening coverage and quality assurance, influence cervical cancer mortality. We aimed to investigate the association between screening coverage, screening quality assurance and cervical cancer mortality among women from countries at different developmental levels.

**Methods:**

We obtained data on age-standardised mortality from cervical cancer from the GLOBOCAN 2020 database; coverage of cervical cancer screening from World Health Organization (WHO) Global Health Observatory; and cervical screening programme settings and quality assurance from the Cancer Screening in Five Continents (CanScreen5) database. We assessed the dependency of cervical cancer age-standardised mortality on screening coverage and quality assurance by simple and multiple regression models. We also used linear regression models to identify factors that improved the screening coverage.

**Results:**

The study included data from 53 countries. Reduced mortality was associated with increased screening programme quality assurance in 22 high-development countries. In 31 low-development countries, screening coverage in women aged 30–49 years was inversely associated with cervical cancer mortality. Political commitment (documentation of the cervical cancer screening policy as law) and financial support (treatment services provided free of charge) positively associated with screening coverage.

**Conclusions:**

Screening programmes need strengthening commensurate with local resources and context. Priority should be given to improving screening coverage through stronger political commitment and financial support in low-development countries, and to ensuring good performance at all levels in high-development countries.

Cervical cancer is one of the gravest threats to women’s lives worldwide and also contributes to the socioeconomic inequality in disease burden [1]. In 2020, around 90% of the 604 127 incident cases and 341 831 deaths due to cervical cancer were estimated in low and middle-income countries (LMIC) [2]. Vaccination against human papilloma virus and screening followed by appropriate treatment of precursor lesions are cost-effective ways to prevent cervical cancer and have become the main strategies in cervical cancer prevention and control [1,3–5]. However, the implementation of effective interventions varies dramatically across countries and regions. This amplifies the global health equality that the fatality rate of cervical cancer is more than 60% in LMICs, and the figure is twice the proportion in high-income countries (HIC). In May 2018, the World Health Organisation (WHO) announced a call for action to eliminate cervical cancer, followed by the 2020 global strategy with a clear target calls for 70% of women to be screened by age 35 and again by age 45 by the year 2030 [6]. If the overall target is achieved by 2030, it is estimated that this would result in 300 000 deaths averted in LMICs [7].

Coverage and quality assurance are essential for screening programmes. Coverage focuses on the breadth of the intervention, by increasing access to screening. An individual-based Monte Carlo simulation model suggested that expanding screening coverage was significant in improving the equity and efficiency of screening, particularly in low-resource settings [8]. Quality assurance emphasises the depth of the intervention, ensuring that screening achieves the expected benefits. European guidelines for quality assurance in cervical cancer screening state that a national, population-based screening programme with good quality assurance is the best practice for early detection of cancer [9]. Achieving high levels of coverage and good quality assurance are therefore the key to reducing the cervical cancer burden and realising the global goal of cervical cancer elimination.

These two significant dimensions of cervical cancer screening vary dramatically across countries at different socioeconomic levels. A global estimation [10] suggested that cervical cancer screening coverage was seven or more times higher in HICs than in LMICs. Several country-level surveys have also highlighted the inequality in screening coverage [11–17]. The status of quality assurance is unclear and hard to monitor, as shown by one Europe-wide survey [18], and is rarely assessed in LMICs. Resource-appropriate prioritisation strategies have not been carefully evaluated, impeding the path to global cervical cancer elimination, especially in LMIC settings with both low screening coverage and unclear quality assurance. Moreover, regarding the high complexity of screening implementation and extreme heterogeneity in programme organisation, an integrated monitoring framework is warranted to evaluate progress towards the goal of cervical cancer elimination and identify next-step priorities to scale up and accelerate the elimination of cervical cancer as a public health problem.

To fill these research gaps, we collected country-level data on screening programmes and health system characteristics from 53 countries and explored the association between screening coverage, quality assurance, and cervical cancer mortality. Our findings provide the largest body of evidence to date to support the development of optimal strategies for resource allocation to improve cervical cancer screening in countries at different levels of development. Our study further introduced an integrated framework to monitor global implementation by combining consecutive evaluation of screening programmes with population-based data and policies and health system capacity.

## METHODS

### Data sources, data collection methods and procedures

We conducted a cross-sectional study design to explore the effects of screening coverage and screening quality assurance on cervical cancer mortality. We developed a new repository using multiple databases from an investigation of cervical cancer service delivery of the Cancer Screening in Five Continents (CanScreen5) project, Global Cancer Observatory (GCO) project, Global Health Observatory (GHO) project, and population-based surveys and cancer registries, as listed in Table S1 in the **Online Supplementary Document**. Countries’ detailed profile of the key variables were listed in Table S2 in the **Online Supplementary Document**. The set of specific indicators was constructed based on the framework for monitoring and evaluation of the WHO cervical cancer elimination strategy, with four domains: population-based data, programme monitoring, policies/programmes and health system capacity, and cross-cutting incidence/mortality. We enriched the domain of programme monitoring according to international consensus on the essential and desirable criteria for an ‘organised’ cancer screening programme by adding related indicators reflecting the policy, initiation, coordination, and financing of the programme; screening tests; invitations and recall facilities; the information system and data collection; and quality assurance of the programme. The integrated framework to monitor global implementation of cervical cancer screening programmes is presented in Figure S1 in the **Online Supplementary Document**.

Data on the age-standardised mortality rate of cervical cancer in women in different countries were retrieved from the International Agency for Research on Cancer (IARC) GLOBOCAN 2020 database [19]. This collects national cancer registry data, and enables a comprehensive assessment of cancer burden worldwide. Data on the coverage of cervical cancer screening were obtained from the WHO GHO [10]. Country-level health system characteristics and socioeconomic status data were obtained from the WHO GHO [20] and World Bank Open Data [21].

Data on cervical screening programme settings and quality assurance were acquired from the IARC CanScreen5 project. This aimed to uniformly collect, analyse, store and disseminate information on the characteristics and performance of cancer screening programmes in different countries. It provided a standard information collection tool and invited representatives from the Ministry of Health, and programme coordinators in different countries to collect and share qualitative and quantitative information on their cervical, breast and colorectal cancer screening programmes. Submitted data go through quality checks and validation by the project secretariat and scientific committee are then disseminated via a web-based open access portal. Detailed information on the CanScreen5 project can be found on the official website (https://canscreen5.iarc.fr/).

Data integrated into the monitoring repository has gone through validation and quality assurance, such as the GHO data set has been checked with the original high-quality population cancer registry data; and questionnaire submitted in CanScreen5 project has been checked by the project secretariat and scientific committee in IARC.

### Definition

Cervical cancer screening coverage was defined as the percentage of women aged 30–49 years screened for cervical cancer in the last year, last three years, last five years and in their lifetime. Cervical cancer screening quality assurance was defined using three indicators: documented standard operating procedure/policy for quality assurance (yes or no), an individual/team/institution responsible for quality assurance (yes or no), and documented performance indicators for quality assurance (yes or no). Countries were classified as having ‘good quality assurance’ if they met all three criteria. Key indicators were selected from the CanScreen5 project to reflect the country-level screening settings, including organisation (dedicated budget for screening programme, nature of documentation of the cervical cancer screening policy, screening tests and treatment services provided free of charge), information system (cancer screening information data linked with population-based cancer registry), and invitation methods (initiatives to create population awareness by the Health Ministry/Health Authority, method of invitation).

Health system data included general characteristics like health finance (domestic general government health expenditure) and health workforce (medical doctors per 10 000 population), and cancer targeted information like ‘existence of national guidelines for the management of cancer’ and ‘existence of national screening programme for cervical cancer’. Country-level economic status was represented by terciles of Gross Domestic Product (GDP) per capita, proportion of women who complete primary-level education and proportion of women with salaried working in the 53 countries included in the analysis. Countries were categorised into low and high development groups using the Human Development Index (HDI) [22], where low development countries were defined as HDI<0.8, and high development countries were defined with HDI≥0.8.

### Statistical analysis

We compared mortality rate of cervical cancer, screening coverage, screening quality assurance strategy, screening programme settings and health system characteristics across countries at different developmental levels. We used the non-parametric Kruskal-Wallis test for continuous variables and the Pearson χ^2^ test for categorical variables.

The dependency of cervical cancer age-standardised mortality on screening coverage and quality assurance was assessed, and further evaluated separately in countries with low and high levels of development. A simple linear regression model was used and the β coefficient was estimated to assess the association between ever-in-lifetime cervical cancer screening coverage in women aged 30–49 years and cervical cancer mortality. A non-parametric Kruskal-Wallis test was used to compare the average cervical cancer mortality in countries with and without good screening quality assurance.

To further explore the dependency of cervical cancer age-standardised mortality on screening coverage and quality assurance, we applied multiple regression models by adding adjusting variables into the model including domestic general government health expenditure (% of GDP), medical doctors (per 10 000 population), existence of national guidelines for the management of cancer (yes or no), existence of national screening programme for cervical cancer (yes or no), GDP per capita terciles, terciles for proportion of women completing primary education, and for proportion of salaried workers who are female, after testing for the normality of the variable distribution and other assumptions. We also used sensitivity analysis to test the robustness of the results, with prevalence of women screened for cervical cancer in the last year, in the last five years, and in the last three years used to represent screening coverage in the multiple regression model.

We decided to further explore the value of screening programme settings in improving screening coverage if coverage was a key factor in reducing cervical cancer mortality. This would use linear regression models with screening coverage as the outcome variable and screening programme settings as the independent variable. Country-level health system characteristics and economic status were included as covariates in multiple regression models.

## RESULTS

### Baseline characteristics

Overall, 53 countries were included in this study, based on the availability of screening programme data. Of these, 31 countries had a low development status (low, middle, and high HDI categories), and 22 countries a high development status (very high HDI category). The baseline characteristics for cervical cancer mortality, screening coverage and quality assurance, screening programme settings are shown in **Table 1** and Figure S2 in the **Online Supplementary Document**. The age-standardised mortality rate of cervical cancer was significantly lower in high development countries (*P* < 0.001). Cervical cancer screening coverage (*P* < 0.001 for ever-in-lifetime percentage; *P* < 0.001 for coverage in previous five years; *P* < 0.001 for coverage in previous three years; *P* < 0.001 for coverage in previous year), quality assurance (*P* = 0.041), documentation of the cervical cancer screening policy as law (*P* = 0.037), linkage of cancer screening information data with population-based cancer registry (*P* < 0.001), domestic general government health expenditure (*P* < 0.001) and number of medical doctors per 10 000 population (*P* < 0.001) significantly increased from countries with low to high levels of development.

**Table 1:** Cervical cancer death rate, screening programme characteristics and settings for 53 countries included in the analysis

Variables	Countries in low, middle, and high HDI categories (n = 31)	Countries in very high HDI category (n = 22)	*P-*value*
**Cervical cancer death rate**			
Age-standardised death rate of cervical cancer in women in 2020			
*Median (quartile)*	12.60 (6.90, 20.60)	2.30 (1.80, 5.20)	<0.001
**Screening coverage**			
Ever in lifetime cervical cancer screening coverage in women aged 30–49 y in 2019			
*Median (quartile)*	31.00 (12.00, 85.00)	92.5 (88.00, 96.00)	<0.001
Cervical cancer screening coverage in the previous five years in women aged 30–49 y in 2019			
*Median (quartile)*	29.00 (10.00, 74.00)	81.00 (74.00, 86.00)	<0.001
Cervical cancer screening coverage in the previous three years in women aged 30–49 y in 2019			
*Median (quartile)*	28.00 (7.00, 61.00)	69.50 (61.00, 76.00)	<0.001
Cervical cancer screening coverage in the previous year in women aged 30–49 y in 2019			
*Median (quartile)*	13.00 (2.00, 30.00)	35.50 (26.00, 43.00)	<0.001
**Screening quality assurance**			
Documented standard operating procedure/policy for quality assurance			
*No*	12 (38.71)	2 (9.09)	0.016
*Yes*	19 (61.29)	20 (90.91)	
An individual/team/institution responsible for quality assurance			
*No*	10 (32.26)	4 (18.18)	0.252
*Yes*	21 (67.74)	18 (81.82)	
Documented performance indicators			
*No*	4 (12.90)	1 (4.55)	0.305
*Yes*	27 (87.10)	21 (95.45)	
Good quality assurance in screening programme†			
*No*	14 (45.16)	4 (18.18)	0.041
*Yes*	17 (54.84)	18 (81.82)	
**Screening programme settings**			
Dedicated budget for screening programme			
*No*	7 (22.58)	2 (9.09)	0.197
*Yes*	24 (77.42)	20 (90.91)	
Nature of documentation of the cervical cancer screening policy			
*Notification or recommendation*	29 (93.55)	16 (72.73)	0.037
*Law*	2 (6.45)	6 (27.27)	
**Screening tests provided free of charge**			
*No*	3 (9.68)	1 (4.55)	0.486
*Yes*	28 (90.32)	21 (95.45)	
Treatment services provided free of charge			
*No*	12 (38.71)	7 (31.82)	<0.001
*Yes*	19 (61.29)	2 (9.09)	
*Unknown*	0 (0.00)	13 (59.09)	
Cancer screening information data are linked with population-based cancer registry			
*No*	29 (93.55)	8 (36.36)	<0.001
*Yes*	2 (6.45)	14 (63.64)	
Initiatives to create population awareness by the Health Ministry/Health Authority‡			
*Less than five kinds of initiatives*	18 (58.06)	18 (81.82)	0.068
*five kinds of initiatives*	13 (41.94)	4 (18.18)	
Method of invitation§			
*No*	23 (74.19)	19 (86.36)	0.282
*Yes*	8 (25.81)	3 (13.64)	
**Health system**			
Existence of national screening programme for cervical cancer			
*No*	4 (12.90)	1 (4.55)	0.305
*Yes*	27 (87.10)	21 (95.45)	
Existence of national guidelines for the management of cancer			
*No*	4 (12.90)	1 (4.55)	0.305
*Yes*	27 (87.10)	21 (95.45)	
Medical doctors (per 10 000 population)			
*Median (quartile)*	7.35 (1.57, 16.65)	34.48 (24.84, 42.25)	<0.001
Domestic general government health expenditure (% of GDP)			
*Median (quartile)*	2.68 (1.56, 3.98)	6.31 (4.86, 7.61)	<0.001
**Socioeconomic status**			
GDP per capita (current USD)			
*Q1 (<4603.34)*	18 (58.06)	0 (0.00)	<0.001
*Q2 (4603.34–20 232.30)*	12 (38.71)	6 (27.27)	
*Q3 (>20 232.30)*	1 (3.23)	16 (72.73)	
Primary completion rate, female			
*Q1 (0–93.48)*	15 (48.39)	3 (13.64)	0.022
*Q2 (93.48–99.00)*	9 (29.03)	8 (36.36)	
*Q3 (>99.00)*	7 (22.58)	11 (50.00)	
Wage and salaried workers, female			
*Q1 (0–51.53)*	18 (58.06)	0 (0.00)	<0.001
*Q2 (51.53–86.85)*	11 (35.48)	7 (31.82)	
*Q3 (>86.85)*	2 (6.45)	15 (68.18)	

GDP – Gross Domestic Product, HDI – Human Development Index

*Differences between continuous variables across HDI categories were tested with the non-parametric Kruskal-Wallis test, and differences between categorical variables were tested with the Pearson χ^2^ test.

†‘Good quality assurance’ was defined as the screening programme with documented standard operating procedure/policy, with an individual/team/institution responsible, and with documented performance indicators for quality assurance.

‡Initiatives to create population awareness included mass media campaign, small media campaign, group education, one-on-one education, dedicate website, and social medial platform.

§Method of invitation included letter, email, SMS, phone call, and home visits by health workers.

### Screening coverage and cervical cancer mortality

**Figure 1:**
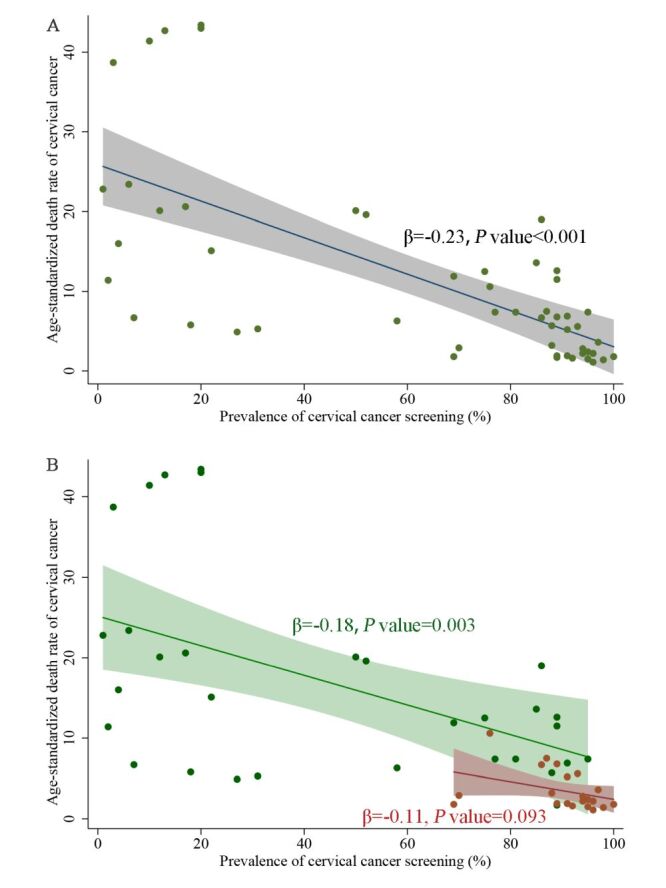
The association between cervical cancer screening coverage and age-standardised cervical cancer mortality in 2020. **Panel A.** The association in 53 countries included in the study. **Panel B.** The association analysis in stratification of country development profiles. The green line refers to the association analysis among 31 countries in low, middle, and high Human Development Index (HDI) categories, and the red line refers to 22 countries in very high HDI categories.

Overall, the cervical cancer screening coverage was associated with lower rate of cervical cancer mortality (β = −0.23, *P* < 0.001) (**Figure 1**, **Table 2**). In stratification of development status, ever-in-lifetime cervical cancer screening coverage in women aged 30–49 years was associated with lower cervical cancer mortality in low development countries (β = −0.18, *P* = 0.003) in the simple linear regression model. This reverse association was also confirmed in the multiple regression analysis, where every one percent increase in screening coverage was associated with a reduction of 0.19 in age-standardised death rate (95% confidential interval (CI) = −0.34, −0.03; *P* = 0.019) in low development countries. However, expanded screening coverage would not further reduce cervical cancer mortality in high-development countries, and the estimated β coefficient between screening coverage and mortality was close to null through multiple regression analysis in these countries (95% CI = −0.12, 0.13; *P* = 0.939).

**Table 2:** Multiple linear regression analysis of screening coverage and screening quality assurance on cervical cancer age-standardised mortality rates

Screening characteristics	Countries included in this study (n = 53)		Countries in low, middle, and high HDI categories (n = 31)		Countries in very high HDI category (n = 22)	
	**β coefficient (95% CI)**	***P-*value**	**β coefficient (95% CI)**	***P-*value**	**β coefficient (95% CI)**	***P*-value**
**Screening coverage**						
Ever in lifetime cervical cancer screening coverage in women aged 30–49 y in 2019	−0.16 (−0.27, −0.05)	0.004	−0.19 (−0.34, −0.03)	0.019	0.00 (−0.12, 0.13)	0.939
**Screening quality assurance**						
Good quality assurance in screening programme						
*No*	Ref	0.062	Ref	0.076	Ref	0.031
*Yes*	5.35 (−0.28, 10.98)		7.75 (−0.87, 16.38)		−2.73 (−5.17, −0.29)	
**Health system**						
Existence of national guidelines for the management of cancer						
*No*	Ref	0.330	Ref	0.721	Ref	0.573
*Yes*	3.90 (−4.09, 11.89)		2.07 (−0.87, 16.38)		−1.12 (−5.33, 3.09)	
Existence of national screening programme for cervical cancer						
*No*	Ref	0.035	Ref	0.160	Ref	0.002
*Yes*	−8.71 (−16.77, −0.65)		−8.02 (−19.46, 3.42)		−6.80 (−10.51, −3.09)	
Medical doctors (per 10 000 population)	−0.07 (−0.29, 0.16)	0.552	−0.26 (−0.68, 0.16)	0.212	−0.01 (−0.08, 0.06)	0.801
Domestic general government health expenditure (% of GDP)	0.75 (−1.11, 2.61)	0.421	3.17 (−0.71, 7.06)	0.104	0.03 (−0.46, 0.52)	0.902
**Socioeconomic status**						
GDP per capita terciles	−4.05 (−9.43, 1.32)	0.136	−2.80 (−12.49, 6.89)	0.554	−2.60 (−4.61, −0.60)	0.015
Primary completion rate, female, terciles	−0.55 (−6.05, 4.95)	0.840	−5.40 (−15.95, 5.16)	0.300	−0.02 (−1.69, 1.64)	0.976
Wage and salaried workers, female, terciles	−3.52 (−6.92, −0.11)	0.043	−5.46 (−10.57, −0.35)	0.037	0.61 (−0.88, 2.10)	0.389

CI – confidential interval, GDP – Gross Domestic Product, HDI – Human Development Index

**Figure 2:**
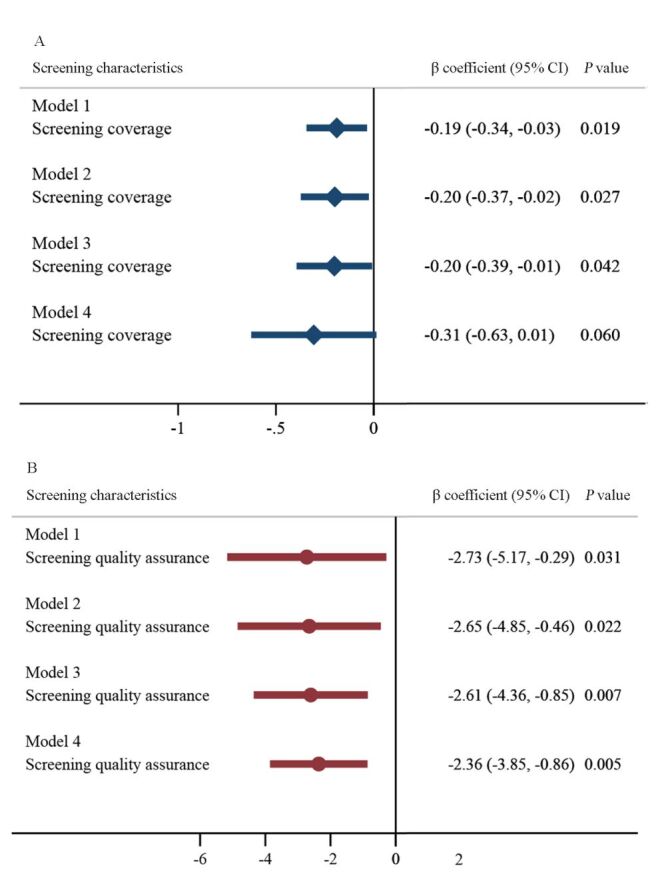
Effect size estimates of screening coverage and quality assurance in the sensitivity analysis. Model 1–4 separately taking screening prevalence in lifetime, in previous five years, in previous three years, and in previous one year as cervical cancer screening coverage. Variables in cervical screening programme quality assurance, health system and socioeconomic status were also included into the multiple linear regression as in **Table 2**. The multiple regression model was conducted in countries in low, middle, and high Human Development Index (HDI) categories, and in countries in very high HDI categories. **Panel A.** The effect size of screening coverage from multiple regression model in 31 countries in low, middle, and high HDI categories. **Panel B.** The effect size of screening programme quality assurance from multiple regression model in 22 countries in very high HDI categories.

The reverse association between screening coverage and mortality was robustly observed in the sensitivity analysis using the percentage of women screened for cervical cancer in the previous five years (β = −0.20, 95% CI = −0.37, −0.02; *P* = 0.027), previous three years (β = −0.20, 95% CI = −0.39, −0.01; *P* = 0.042), and the previous year (β = −0.31, 95% CI = −0.63, 0.01; *P* = 0.060) for cervical cancer screening coverage (**Figure 2**, Table S3 in the **Online Supplementary Document**).

### Screening quality assurance and cervical cancer mortality

In 22 highly developed countries, lower mortality was associated with increased screening programme quality assurance (Figure S3 in the **Online Supplementary Document**; *P* = 0.022 for documented standard operating procedure; *P* = 0.002 for responsible team; *P* = 0.097 for documented performance indicators; *P* = 0.002 for synthetic quality assurance indicator). In 31 low-development countries, there was no significant difference in the age-standardised mortality of cervical cancer by screening quality assurance factors (*P* = 0.162 for documented standard operating procedure; *P* = 0.800 for responsible team; *P* = 0.082 for documented performance indicators; *P* = 0.275 for synthetic quality assurance indicator). In the multiple regression analysis including health system characteristics and socioeconomic status as covariates, good quality assurance was significantly related to lower cervical cancer mortality in high-development countries (β = −2.73, 95% CI = −5.17, −0.29; *P* = 0.031 (**Table 2**). However, this association was not seen in low-development countries (β = 7.75, 95% CI = −0.87, 16.38; *P* = 0.076).

### Screening programme settings and cervical cancer screening coverage

Given the significant association between screening coverage and cervical cancer mortality in low development countries, a further analysis was conducted to identify the key factors in screening programmes that were associated with higher screening coverage (**Table 3**). The simple regression model showed that free-of-charge treatment services (β = 36.15, 95% CI = 13.01, 59.30; *P* = 0.003), and linkage of cancer screening information data and a population-based cancer registry (β = 50.52, 95% CI = 0.72, 100.31; *P* = 0.047) were significantly associated with high-level screening coverage in countries in low, middle and high HDI categories. Multiple regression analysis suggested that free-of-charge treatment services (β = 22.59, 95% CI = 0.22, 44.96; *P* = 0.048) and documentation of the cervical cancer screening policy as law (β = 46.22, 95% CI = 1.50, 90.93; *P* = 0.044) were crucial to improving cervical cancer screening coverage in women aged 30–49 years.

**Table 3:** Simple and multiple linear regression analysis of the effects of screening programme settings on cervical cancer coverage in 31 countries in low, middle, and high Human Development Index (HDI) categories

	Simple regression model		Multiple regression model	
	**β coefficient (95% CI)**	***P*-value**	**β coefficient (95% CI)**	***P*-value**
**Screening programme characteristics**				
Dedicated budget for screening programme				
*No*	Ref	0.449	Ref	0.625
*Yes*	11.67 (−19.38, 42.70)		−6.46 (−33.90, 20.99)	
Nature of documentation of the cervical cancer screening policy				
*Notification or recommendation*	Ref	0.074	Ref	0.044
*Law*	45.71 (−4.75, 96.17)		46.22 (1.50, 90.93)	
Screening tests provided free of charge				
*No*	Ref	0.707	Ref	0.583
*Yes*	8.20 (−36.03, 52.43)		10.62 (−29.59, 50.83)	
Treatment services provided free of charge				
*No*	Ref	0.003	Ref	0.048
*Yes*	36.15 (13.01, 59.30)		22.59 (0.22, 44.96)	
Cancer screening information data are linked with population-based cancer registry				
*No*	Ref	0.047	Ref	0.187
*Yes*	50.52 (0.72, 100.31)		32.48 (−17.47, 82.44)	
Initiatives to create population awareness by the Health Ministry/Health authority				
*Less than five kinds of initiatives*	Ref	0.675	Ref	0.876
*five kinds of initiatives*	5.48 (−21.01, 31.96)		1.77 (−21.98, 25.52)	
Invitation				
*No*	Ref	0.085	Ref	0.700
*Yes*	24.78 (−3.67, 53.22)		−5.62 (−35.97, 24.72)	
**Health system characteristics**				
Existence of national guidelines for the management of cancer				
*No*	Ref	0.057	Ref	0.914
*Yes*	35.58 (−1.11, 72.28)		1.73 (−31.60, 35.05)	
Existence of national screening programme for cervical cancer				
*No*	Ref	0.259	Ref	0.555
*Yes*	21.52 (−16.72, 59.76)		−9.68 (−43.71, 24.35)	
Medical doctors (per 10 000 population)	1.19 (0.56, 1.82)	0.001	0.39 (−0.87, 1.64)	0.524
Domestic general government health expenditure (% of GDP)	11.09 (7.03, 15.15)	<0.001	5.94 (−4.00, 1.64)	0.224
**Socioeconomic status**				
GDP per capita terciles	37.37 (18.68, 56.05)	<0.001	20.19 (−5.68, 46.07)	0.118
Primary completion rate, female, terciles	10.93 (−4.88, 26.74)	0.168	−16.18 (−44.97, 12.60)	0.251
Wage and salaried workers, female, terciles	31.67 (14.09, 49.25)	0.001	5.61 (−11.67, 22.89)	0.501

CI – confidential interval, GDP – Gross Domestic Product, HDI – Human Development Index

## DISCUSSION

To the best of our knowledge, this study is the first to comprehensively evaluate the role of improving screening coverage and enhancing quality assurance in reduction of cervical cancer mortality, by collecting programmatic settings and health system characteristics from 53 countries. The inverse relationship between coverage and cervical cancer mortality was significant in countries with low levels of development, and programme quality assurance appears to play a stronger role in reducing cervical cancer mortality in countries with high levels of development. These findings highlight the importance of a resource-appropriate screening improvement strategy, and suggest ways to improve global cervical outcomes by identifying priorities for screening programme strengthening in countries at different developmental levels.

### Identifying the key strategy to reduce cervical cancer mortality in LMICs and HICs

Screening coverage is a major constraint in low-development countries. Low screening coverage will increase the risk of individual women dying from invasive cancers. This will prevent screening programmes from having the desired impact on cervical cancer incidence and mortality. Among the 31 countries with low, medium, and high HDI levels included in this study, only 31% of women aged 30–49 years had received cervical cancer screening in their lifetime, which was about one-third of the 92.5% coverage rate in countries with very high HDI. Similarly, a global descriptive study collected data from population-based surveys in 57 countries and estimated a crude coverage rate of 44.7% in developing countries, about half the percentage of developed countries [23]. Our results show that cervical cancer mortality was inversely associated with screening coverage i.e. for each percent increase in screening coverage, the age-standardised mortality rate for cervical cancer decreased by 0.19 in low-development countries. A Mexican study reported a similar inverse relationship, finding that the decrease in cervical cancer mortality was proportional to increasing Papanicolaou (a type of technology in cervical cancer screening) coverage and that the mortality rate decreased by 0.07 for each unit increase in screening coverage [24]. Ensuring adequate coverage is therefore fundamental to cervical cancer elimination, and low-development countries should concentrate on improving screening coverage.

Coverage of cervical cancer screening could be improved by strengthened screening programmatic settings. We found that political commitment, documentation of the cervical cancer screening policy as law (β = 46.22, 95% CI = 1.50, 90.93; *P* = 0.044), and financial support, treatment services provided free of charge (β = 22.59, 95% CI = 0.22, 44.96; *P* = 0.048), resulted in higher screening coverage. Country-level analysis also showed that health system and programmatic settings could remove obstacles in the way of screening implementation. In Bamako, Mali, a coverage-increasing strategy ‘Weekend70 programme’ showed good performance at individual, interpersonal, organisational, community and political levels [25]. Financial cost was a structural barrier to screening participation [26], and adequate investment in funding for screening programmes appears to be positively associated with cervical cancer screening uptake [27]. We found that free-of-charge treatment had a stronger effect on improving screening participation than free-of-charge screening services. This implies that the cost of treatment for precancerous lesions was the main concern preventing women from participating in screening programmes. Future financial support could be weighted more towards precancer treatment to remove concerns. Integrating screening into the existing health system and considering the overall flow of cervical cancer management is essential to the sustainable scaling-up of screening programmes. Several trials and meta-analyses have shown that awareness-raising initiatives and health education are also valuable ways to improve screening coverage, especially in LMICs [28–30]. We found a positive but nonsignificant association between awareness campaigns and screening coverage, which may be because of the implementation diversity across countries and the limited statistical power of this analysis.

For high-development countries that already have adequate screening coverage, the priority should be to strengthen quality assurance of screening programmes to further decrease cervical cancer mortality. We found that very high HDI countries with good quality assurance in screening programmes had lower cervical cancer mortality. European and North American countries have most strongly advocated for quality assurance and quality improvement in screening. The first European guidelines for quality assurance of cervical cancer screening were published in 1993, outlining principles for organising screening, monitoring its impact and ensuring the quality of screening tests. The European guidelines for quality assurance in cervical cancer screening emphasise that all-level quality assurance in organised population-based screening programmes is critical to the success of cervical cancer screening programmes [9]. The United States also focuses on quality assurance in screening programmes. The National Breast and Cervical Cancer Early Detection Programme was initiated in 1991 to identify problems across screening programmes and systematically correct these through quality improvement, related experience showed that continuous monitoring of the quality assurance of screening projects has significantly improved the quality of the screening service and patient outcomes [31]. Our findings, together with experience from Europe and the United States, suggest that strong quality assurance is crucial to ensuring that services meet the requisite standards and guaranteeing that screening programmes achieve the desired impact of reducing incidence and mortality. Improving quality assurance in screening programmes should therefore be a priority in high-development countries.

### Policy implications for global monitoring and national practices

The Seventy-third World Health Assembly adopted the global strategy to accelerate the elimination of cervical cancer as a public health problem and its associated goals and targets for the period 2020–2030. The WHO was requested to prepare regular updates on the progress towards achieving the 2030 targets and to provide technical support on best practices and recommendations for further acceleration towards elimination. To facilitate monitoring and evaluation of the WHO cervical cancer elimination strategy, we presented an integrated framework, synthesising the settings of screening programmes with the overall framework, to provide a series of population-based and programme-specific indicators and construct a data repository based on several closely connected WHO- and IARC-initiated projects. This integrated framework has policy and practice implications for the global monitoring and evaluation of cervical cancer screening implementation and identifying priority areas of action. Analysis of the repository based on the framework could yield evidence-based interventions and support countries in developing national screening implementation plans. It emphasises the need to focus on the organisation and performance of screening programmes and the complex interplay with health systems when monitoring and supervising the global cervical cancer prevention and control strategy.

The integrated framework recognises the organisation and supportive policies in cervical cancer screening programme implementation by setting a series of performance indicators across the whole screening continuum, instead of only focusing on the positive screening test rate and treatment rate in the existing monitoring framework. The performance of the screening programme is fundamentally crucial to reducing the cervical cancer burden; however, improved screening programme performance can only be accomplished through suitable organisation of the programmes, coupled with strengthened downstream diagnostic and treatment services, an effective referral system, and a well-prepared health system. Thus, this integrated framework to collect multi-level information, including programme organisation, performance, and related population-level outcomes, cannot only yield a comprehensive profile of global implementation status and progress in the cervical cancer screening strategy but also provide a rational mechanism to support continuous quality improvement of services, and ultimately, accomplishment of the global goal to eliminate cervical cancer.

Collection, management, and analysis based on the integrated multi-source repositories from WHO- and IARC-initiated projects can provide policymakers, programme managers, and service providers with the information needed to make informed decisions, improve programmes, and gain the desired population-based outcomes. As represented by the first case study, we identified the next-step priorities for implementation in cervical cancer screening; that is, to scale-up the screening coverage for LMICs and strengthen quality control measures for HICs, according to a global comparative analysis. Analysis based on the integrated framework could also help to summarise the best practices in response to divergent health systems, enabling policymakers and programme managers to identify gaps and take specific actions. For example, LMICs with insufficient screening coverage may consider increasing political commitment or financial support to leverage coverage, as illustrated by the findings of the second case study. This repository according to the integrated framework could also provide evidence-based best practices regarding establishment of a referral mechanism between screening programmes and clinical treatment services, financial support to maximise benefits of the continuum of cervical cancer prevention and control, and dynamic adjustment of national plans and practices based on a regularly updated longitudinal database.

### Strengths and limitations

This study has several strengths. First, data from screening programmes were submitted from country-level programme coordinators and validated by the CanScreen5 secretariat, and the quality is therefore assured. Second, the results of the cross-country study systematically demonstrated the role of screening coverage and quality assurance in reducing cervical cancer mortality, and identified possible priorities for screening improvement strategies in low and high development countries.

However, the study also has some limitations. First, it only included 53 countries with complete information, which may limit the statistical power to identify influencing factors in screening programmes. However, it is the largest body of evidence to date used to examine the association between screening coverage, quality assurance and cervical cancer mortality. Second, duration of screening programmes was not included in current analysis due to a proportion of missing values in the CanScreen5 data set. In this analysis, screening coverage partly represented the role of programme duration (Pearson correlation coefficient = 0.50, *P* < 0.001). The independent association between programme timing on cervical cancer mortality could be further explored in any future analysis using panel data model.

## CONCLUSIONS

Screening coverage and quality assurance, are associated with reducing cervical cancer mortality. Improving screening coverage through strengthened political commitment and financial support should be the top priority for low-development countries, and high-development countries should focus on ensuring good performance of screening programmes at all levels. Analysis of the repository according to the integrated framework could facilitate monitoring and evaluation of the WHO cervical cancer elimination strategy and provide technical support to country-level policymakers and screening programme managers to identify priorities for action towards achieving the desired population-based outcomes.

## Additional material


Online Supplementary Document
