# Supplementary material for: Effects of screening coverage and screening quality assurance on cervical cancer mortality: Implication for integrated framework to monitor global implementation of cervical cancer screening programmes
Source: J Glob Health. 2024 Oct 25;14:04189. doi: 10.7189/jogh.14.04189 (PMC11503509; doi:10.7189/jogh.14.04189)
Supplement: Online Supplementary Document [file jogh-14-04189-s001.pdf]

**Effects of screening coverage and screening quality assurance on cervical cancer mortality: Implication for integrated framework to monitor global implementation of cervical cancer screening programmes**

**Supplemental Materials**

**Table S1.** Data source of the variables included in the analysis.

**Table S2.** Country profile of the key variables for 53 countries included into analysis.

**Table S3.** Sensitivity analysis of the effects of screening program settings on cervical cancer coverage using different screening coverage indicators.

**Figure S1**. Integrated framework for monitoring and evaluation of the WHO cervical cancer elimination strategy.

**Figure S2.** HDI category (**Panel A**), age-standardized death rate of cervical cancer in 2020 (**Panel B**), ever in lifetime cervical cancer screening coverage (**Panel C**), and cervical cancer screening quality assurance (**Panel D**) in 53 countries included into current study.

**Figure S3.** The association between cervical cancer screening quality assurance and age-standardized cervical cancer death rate in 2020, by country development profiles. **Panel A.** Quality assurance component 1: standard operating procedure; **Panel B.** Quality assurance component 2: responsible team for QA; **Panel C.** Quality assurance component 3: performance indicator; **Panel D.** Synthesis quality assurance indicator. The P values were calculated through non-parametric Kruskal-Wallis test.

**Table S1.** Data source of the variables included in the analysis.

| Variable | Data source |
|---|---|
| Age-standardized death rate of cervical cancer, female | WHO Global Cancer Observatory (GCO, https://gco.iarc.fr/) |
| GDP per capita (current US$) | World Bank Open Data (https://data.worldbank.org/) |
| Primary completion rate, female | World Bank Open Data (https://data.worldbank.org/) |
| Wage and salaried workers, female | World Bank Open Data (https://data.worldbank.org/) |
| Existence of national guidelines for the management of cancer | WHO Global Health Observatory (GHO, https://www.who.int/data/gho |
| Existence of national screening program for cervical cancer | WHO Global Health Observatory (GHO, https://www.who.int/data/gho |
| Medical doctors (per 10 000 population) | WHO Global Health Observatory (GHO, https://www.who.int/data/gho |
| Domestic general government health expenditure (% of GDP) | World Bank Open Data (https://data.worldbank.org/) |
| Dedicated budget for screening programme | Cancer Screening in Five Continents (CanScreen5, https://canscreen5.iarc.fr/) |
| Nature of documentation of the cervical cancer screening policy | Cancer Screening in Five Continents (CanScreen5, https://canscreen5.iarc.fr/) |
| Ever in lifetime cervical cancer screening coverage in women aged 30–49 years in 2019 | WHO Global Health Observatory (GHO, https://www.who.int/data/gho |
| Cervical cancer screening coverage in the previous 5 years in women aged 30–49 years in 2019 | WHO Global Health Observatory (GHO, https://www.who.int/data/gho |
| Cervical cancer screening coverage in the previous 3 years in women aged 30–49 years in 2019 | WHO Global Health Observatory (GHO, https://www.who.int/data/gho |
| Cervical cancer screening coverage in the previous year in women aged 30–49 years in 2019 | WHO Global Health Observatory (GHO, https://www.who.int/data/gho |

| | |
|---|---|
| Documented standard operating procedure/policy for quality assurance | Cancer Screening in Five Continents (CanScreen5, https://canscreen5.iarc.fr/) |
| An individual/team/institution responsible for quality assurance | Cancer Screening in Five Continents (CanScreen5, https://canscreen5.iarc.fr/) |
| Documented performance indicators | Cancer Screening in Five Continents (CanScreen5, https://canscreen5.iarc.fr/) |
| Screening tests provided free of charge | Cancer Screening in Five Continents (CanScreen5, https://canscreen5.iarc.fr/) |
| Treatment services provided free of charge | Cancer Screening in Five Continents (CanScreen5, https://canscreen5.iarc.fr/) |
| Cancer screening information data is linked with population-based cancer registry | Cancer Screening in Five Continents (CanScreen5, https://canscreen5.iarc.fr/) |
| Initiatives to create population awareness by the Health Ministry/Health Authority | Cancer Screening in Five Continents (CanScreen5, https://canscreen5.iarc.fr/) |
| Method of invitation | Cancer Screening in Five Continents (CanScreen5, https://canscreen5.iarc.fr/) |

**Table S2.** Country profile of the key variables for 53 countries included into analysis.

| Country name | HDI index | Age-standardized death rate of cervical cancer in 2020 | Cervical cancer screening coverage (%) | Domestic general government health expenditure (% of GDP) | Primary completion rate, female |
|---|---|---|---|---|---|
| Low development countries | | | | | |
| Mozambique | 0.456 | 38.7 | 3 | 40.2 | 55.0204 |
| Ethiopia | 0.485 | 16 | 4 | 24.2 | 65.3713 |
| United Republic of Tanzania | 0.529 | 42.7 | 13 | 36.8 | 72.0052 |
| CÃ´te d'Ivoire | 0.538 | 22.8 | 1 | 72 | 82.1693 |
| Rwanda | 0.543 | 20.1 | 12 | 58.3 | 104.129 |
| Uganda | 0.544 | 41.4 | 10 | 43.1 | 53.8109 |
| Cameroon | 0.563 | 23.4 | 6 | 54.1 | 62.365 |
| Zimbabwe | 0.571 | 43 | 20 | 140.3 | 90.9025 |
| Zambia | 0.584 | 43.4 | 20 | 76 | 78.5825 |
| Kenya | 0.601 | 20.6 | 17 | 88.4 | 100.085 |
| Bangladesh | 0.632 | 6.7 | 7 | 41.9 | 71.7645 |
| Honduras | 0.634 | 12.5 | 75 | 176.2 | 79.1248 |
| India | 0.645 | 11.4 | 2 | 72.8 | 96.0652 |
| Nicaragua | 0.66 | 12.6 | 89 | 173.8 | 88.8012 |
| Guatemala | 0.663 | 11.9 | 69 | 259.6 | 81.5214 |
| El Salvador | 0.673 | 7.4 | 95 | 288.5 | 100.486 |
| Guyana | 0.682 | 15.1 | 22 | 295.5 | 95.5467 |
| Morocco | 0.686 | 5.8 | 18 | 174.5 | 100.748 |
| South Africa | 0.709 | 19.6 | 52 | 525.9 | 93.2365 |

| | | | | | |
|---|---|---|---|---|---|
| Paraguay | 0.728 | 19 | 86 | 400.4 | 88.8148 |
| Jamaica | 0.734 | 13.6 | 85 | 321 | 76.5304 |
| Botswana | 0.735 | 20.1 | 50 | 483 | 95.1415 |
| China | 0.761 | 5.3 | 31 | 501 | 97.6633 |
| Brazil | 0.765 | 6.3 | 58 | 848.4 | 99 |
| Colombia | 0.767 | 7.4 | 81 | 513.1 | 103.778 |
| Peru | 0.777 | 11.5 | 89 | 369 | 95.9527 |
| Thailand | 0.777 | 7.4 | 77 | 275.9 | 95.3832 |
| Mexico | 0.779 | 5.7 | 88 | 519.6 | 103.25 |
| Sri Lanka | 0.782 | 4.9 | 27 | 157.5 | 97.9032 |
| Cuba | 0.783 | 6.9 | 91 | 987 | 95.5184 |
| High development countries | | | | | |
| Bahamas | 0.814 | 10.6 | 76 | 2013.9 | 93.4846 |
| Panama | 0.815 | 7.5 | 87 | 1131.6 | 86.8466 |
| Uruguay | 0.817 | 5.6 | 93 | 1590 | 104.132 |
| Turkey | 0.82 | 2.2 | 96 | 389.9 | 90.4399 |
| Chile | 0.851 | 5.2 | 91 | 1455.6 | 97.694 |
| Portugal | 0.864 | 3.2 | 88 | 2215.1 | 99.1559 |
| Latvia | 0.866 | 6.8 | 89 | 1101.5 | 98.6389 |
| Lithuania | 0.882 | 6.7 | 86 | 1249.2 | 104.314 |
| Italy | 0.892 | 1.6 | 92 | 2989 | 97.3896 |
| Czechia | 0.9 | 3.6 | 97 | 1765.6 | 102.368 |
| France | 0.901 | 2.2 | 94 | 4690 | 95 |
| Republic of Korea | 0.916 | 1.8 | 69 | 2542.8 | 105.424 |
| Slovenia | 0.917 | 2.4 | 95 | 2169.6 | 99.0018 |

| | | | | | |
|---|---|---|---|---|---|
| Japan | 0.919 | 2.9 | 70 | 4266.6 | 99 |
| Canada | 0.929 | 1.9 | 91 | 4994.9 | 97.8916 |
| United Kingdom | 0.932 | 1.9 | 89 | 4315.4 | 101.497 |
| Finland | 0.938 | 1.1 | 96 | 4515.7 | 100.982 |
| Denmark | 0.94 | 2.2 | 96 | 6216.7 | 101.599 |
| Australia | 0.944 | 1.5 | 95 | 5425.3 | |
| Netherlands | 0.944 | 1.4 | 98 | 5306.5 | 95 |
| Sweden | 0.945 | 1.8 | 100 | 5981.7 | 104.666 |
| Ireland | 0.955 | 2.8 | 94 | 5489 | 102.422 |
| Norway | 0.957 | 1.7 | 89 | 8239.1 | 100.293 |

**Table S3.** Sensitivity analysis of the effects of screening program settings on cervical cancer coverage using different screening coverage indicators.

| Screening characteristics | Cervical cancer screening coverage (5 years) | | Cervical cancer screening coverage (3 years) | | Cervical cancer screening coverage (1 year) | |
|---|---|---|---|---|---|---|
| | **β coefficient (95% CI)** | ***P* value** | **β coefficient (95% CI)** | ***P* value** | **β coefficient (95% CI)** | ***P* value** |
| **Screening program characteristics** | | | | | | |
| Dedicated budget for screening programme | | | | | | |
| No | Ref | 0.622 | Ref | 0.619 | Ref | 0.429 |
| Yes | -5.67 (-29.59, 18.24) | | -4.93 (-25.58, 15.72) | | -5.00 (-18.04, 8.05) | |
| Nature of documentation of the cervical cancer screening policy | | | | | | |

| | | | | | | |
|---|---|---|---|---|---|---|
| Notification or Recommendation | Ref | 0.019 | Ref | 0.007 | Ref | 0.026 |
| Law | 48.01 (9.04, 8.97) | | 49.48 (15.83, 83.13) | | 24.61 (3.36, 45.87) | |
| Screening tests provided free of charge | | | | | | |
| No | Ref | 0.507 | Ref | 0.335 | Ref | 0.223 |
| Yes | 11.22 (-2.81, 46.26) | | 14.19 (-16.07, 44.45) | | 11.44 (-7.68, 30.55) | |
| Treatment services provided free of charge | | | | | | |
| No | Ref | 0.057 | Ref | 0.074 | Ref | 0.071 |
| Yes | 18.87 (-0.62, 38.37) | | 15.2 (-1.63, 32.04) | | 9.7 (-0.93, 20.34) | |
| Cancer screening information data is linked with population-based cancer registry | | | | | | |
| No | Ref | 0.158 | Ref | 0.13 | Ref | 0.398 |
| Yes | 30.38 (-13.15, 73.91) | | 28.28 (-9.31, 65.87) | | 9.73 (-14.02, 33.47) | |
| Initiatives to create population awareness by the Health Ministry/Health Authority [c] | | | | | | |
| Less than 5 kinds of initiatives | Ref | 0.906 | Ref | 0.695 | Ref | 0.185 |
| 5 kinds of initiatives | 1.18 (-19.52, 21.87) | | 3.37 (-14.5, 21.24) | | 7.37 (-3.91, 18.66) | |
| Method of invitation [d] | | | | | | |
| No | Ref | 0.757 | Ref | 0.857 | Ref | 0.327 |
| Yes | -3.93 (-30.37, 22.51) | | -1.98 (-24.81, 20.86) | | 6.88 (-7.55, 21.3) | |
| **Health system characteristics** | | | | | | |

| | | | | | | |
|---|---|---|---|---|---|---|
| Existence of national guidelines for the management of cancer | | | | | | |
| No | Ref | 0.943 | Ref | 0.89 | Ref | 0.281 |
| Yes | 0.99 (-28.05, 30.03) | | -1.67 (-26.75, 23.41) | | -8.34 (-24.18, 7.5) | |
| Existence of national screening program for cervical cancer | | | | | | |
| No | Ref | 0.490 | Ref | 0.354 | Ref | 0.169 |
| Yes | -9.88 (-39.54, 19.77) | | -11.52 (-37.13, 14.09) | | -11.01 (-27.18, 5.17) | |
| Medical doctors (per 10 000 population) | 0.47 (-0.62, 1.56) | 0.375 | 0.58 (-0.37, 1.52) | 0.213 | 0.25 (-0.35, 0.84) | 0.392 |
| Domestic general government health expenditure (% of GDP) | 5.11 (-3.55, 13.77) | 0.229 | 3.85 (-3.63, 11.33) | 0.291 | 3.76 (-0.97, 8.48) | 0.111 |
| **Socioeconomic status** | | | | | | |
| GDP per capita terciles | 17.02 (-5.52, 39.57) | 0.129 | 13.8 (-5.67, 33.28) | 0.152 | 1.48 (-10.82, 13.78) | 0.802 |
| Primary completion rate, female, terciles | -16.29 (-41.38, 8.79) | 0.188 | -14.24 (-35.9, 7.42) | 0.182 | -7.95 (-21.63, 5.73) | 0.236 |
| Wage and salaried workers, female, terciles | 5.24 (-9.82, 20.29) | 0.472 | 4.93 (-8.07, 17.93) | 0.433 | 4.17 (-4.05, 12.38) | 0.298 |

## FRAMEWORK FOR MONITORING AND EVALUATION OF THE WHO CERVICAL CANCER ELIMINATION STRATEGY

<table>
<tr><th></th><th colspan="2">Primary prevention</th><th colspan="2">Secondary prevention</th><th colspan="2">Tertiary prevention</th></tr>
<tr><td>2030 targets</td><td colspan="2">HPV vaccination and health promotion<br>90%<br>of girls fully vaccinated with HPV vaccine by 15 years of age</td><td colspan="2">Screening and pre-cancer treatment<br>70%<br>of women screened using a high-performance test by 35 and again by 45 years of age</td><td colspan="2">Treatment and supportive care<br>90%<br>of women identified with a cervical disease are treated (90% of women with precancer treated and 90% of women with invasive cancer managed)</td></tr>
<tr><td>Population-based data</td><td colspan="2">• HPV prevalence<br>• HIV prevalence<br>• Tobacco use prevalence<br>• Condom use at last high-risk sex prevalence</td><td colspan="2">• Screening coverage, including with a high-performance test<br>• Cervical pre-cancer incidence</td><td colspan="2">• Cervical cancer survival<br>• Cervical cancer mortality-to-incidence ratio</td></tr>
<tr><td>Programme monitoring</td><td colspan="2">• Commitment & Demand<br>• Coverage & Equity<br>• Life Course & Integration<br>• Outbreaks & Emergencies<br>• Supply & Sustainability<br>• Research & Innovation</td><td colspan="2">• Programme policy, initiation, coordination, and financing<br>• Screening test<br>• Invitations and recall facilities<br>• Information system and data collection<br>• Quality assurance of programme</td><td colspan="2">• Stage at diagnosis<br>• Invasive cervical cancer treatment coverage<br>• Quality of Invasive cervical cancer treatment<br>• Palliative care<br>• Quality of palliative care</td></tr>
<tr><td rowspan="3">Policies/ Programmes and health system capacity</td><td colspan="2">• HPV vaccine in National Immunization Programme<br>• HPV vaccine supply and availability<br>• HPV vaccine cost</td><td colspan="2">• Availability of national cervical cancer screening program<br>• Availability of pre-cancer treatment<br>• HPV test availability in PHC</td><td colspan="2">• Availability of guidelines for the management of women with cervical disease, including high-risk groups<br>• Availability of treatment - pathology/ surgery/ chemotherapy/ radiotherapy<br>• Availability of specialized medical staff<br>• Availability of palliative care medications</td></tr>
<tr><td colspan="2"></td><td colspan="4">• Referral pathway for screen-positive women (linkage to treatment)</td></tr>
<tr><td colspan="6">• Availability and quality of surveillance system (population-based surveys, pre-cancer or screening registries, population-based cancer registries, facility-based reporting system, health facility surveys, civil registration and vital statistics system)</td></tr>
<tr><td>Cross cutting Incidence and mortality</td><td colspan="2">Cumulative risk of cervical cancer</td><td>Cervical cancer incidence</td><td colspan="2">Cervical cancer mortality</td><td>Premature mortality from cervical cancer</td></tr>
</table>

**Figure S1**. Integrated framework for monitoring and evaluation of the WHO cervical cancer elimination strategy.

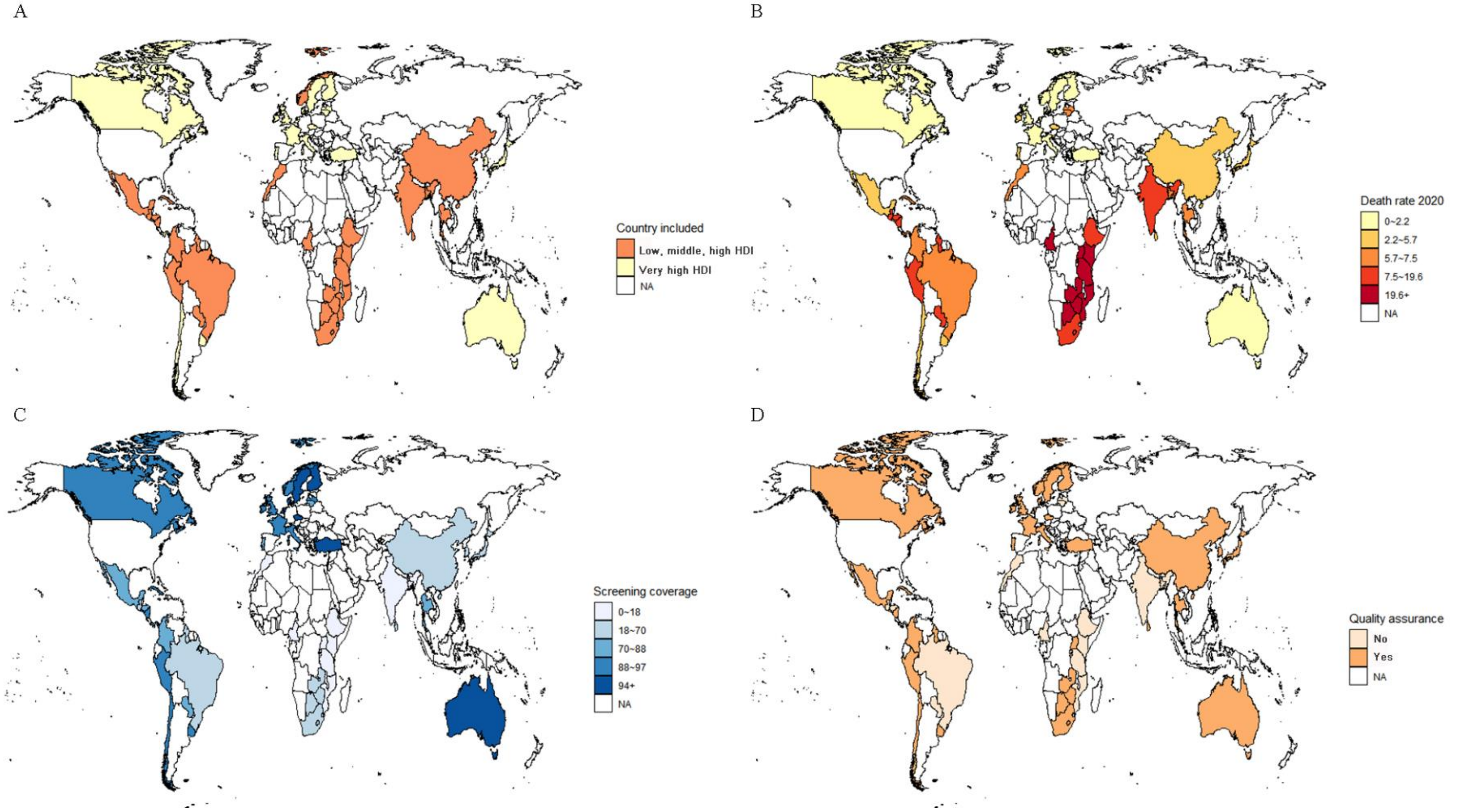


**Figure S2.** HDI category (**Panel A**), age-standardized death rate of cervical cancer in 2020 (**Panel B**), ever in lifetime cervical cancer screening coverage (**Panel C**), and cervical cancer screening quality assurance (**Panel D**) in 53 countries included into current study.

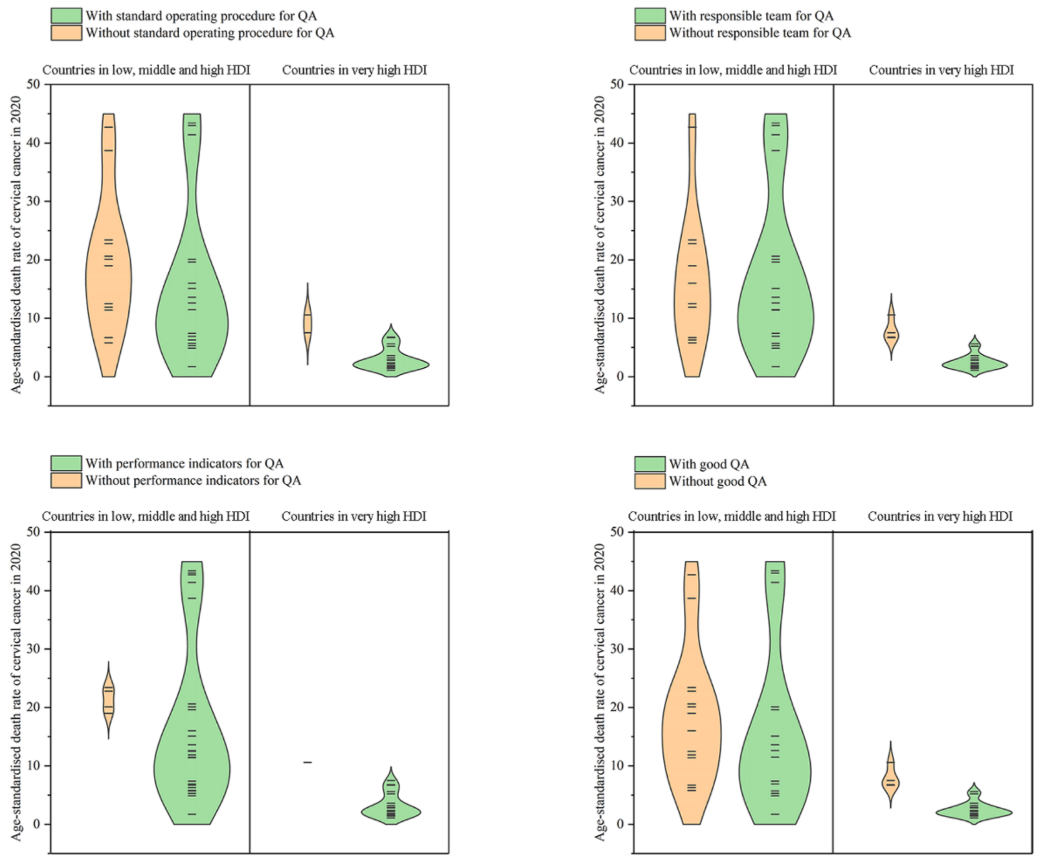


**Figure S3.** The association between cervical cancer screening quality assurance and age-standardized cervical cancer death rate in 2020, by country development profiles. **Panel A.** Quality assurance component 1: standard operating procedure; **Panel B.** Quality assurance component 2: responsible team for QA; **Panel C.** Quality assurance component 3: performance indicator; **Panel D.** Synthesis quality assurance indicator. The P values were calculated through non-parametric Kruskal-Wallis test.
